# Conversion Kinetics and Ionic Conductivity in Na-β”-Alumina + YSZ (Naβ”AY) Sodium Solid Electrolyte via Vapor Phase Conversion Process

**DOI:** 10.3390/membranes12060567

**Published:** 2022-05-30

**Authors:** Liangzhu Zhu, Anil V. Virkar

**Affiliations:** 1 Materials Science & Engineering Department, University of Utah, Salt Lake City, UT 84112, USA; 2 Key Laboratory of Advanced Fuel Cells and Electrolyzers Technology of Zhejiang Province, Ningbo Institute of Materials Technology and Engineering, Chinese Academy of Sciences, Ningbo 315201, China

**Keywords:** sodium β”-alumina, Naβ”AY, sodium electrolyte, sodium solid-state battery, vapor phase process, sodium batteries

## Abstract

Sodium ion batteries have been receiving increasing attention and may see potential revival in the near future, particularly in large-scale grid energy storage coupling with wind and solar power generation, due to the abundant sodium resources, low cost, and sufficiently high energy density. Among the known sodium ion conductors, the Na-β”-alumina electrolyte remains highly attractive because of its high ionic conductivity. This study focuses on the vapor phase synthesis of a Na-β”-Alumina + YSZ (Naβ”AY) composite sodium electrolyte, which has higher mechanical strength and stability than conventional single phase β”-Alumina. The objectives are the measurement of conversion kinetics through a newly developed weight-gain based model and the determination of sodium ionic conductivity in the composite electrolyte. Starting samples contained ~70 vol% α-Alumina and ~30 vol% YSZ (3 mol% Y_2_O_3_ stabilized Zirconia) with and without a thin alumina surface layer made by sintering in air at 1600 °C. The sintered samples were placed in a powder of Na-β”-alumina and heat-treated at 1250 °C for various periods. Sample dimensions and weight were measured as a function of heat treatment time. The conversion of α-Alumina in the α-Alumina + YSZ composite into Naβ”AY occurred by coupled diffusion of sodium ions through Na-β”-alumina and of oxygen ions through YSZ, effectively diffusing Na_2_O. From the analysis of the time dependence of sample mass and dimensions, the effective diffusion coefficient of Na_2_O through the sample, $D_{eff}$, was estimated to be 1.74 × 10^−7^ cm^2^ s^−1^, and the effective interface transfer parameter, $k_{eff}$, was estimated as 2.33 × 10^−6^ cm s^−1^. By depositing a thin alumina coating layer on top of the bulk composite, the chemical diffusion coefficient of oxygen through single phase Na-β”-alumina was estimated as 4.35 × 10^−10^ cm^2^ s^−1^. An AC impedance measurement was performed on a fully converted Naβ”AY composite, and the conductivity of the composite electrolyte was 1.3 × 10^−1^ S cm^−1^ at 300 °C and 1.6 × 10^−3^ S cm^−1^ at 25 °C, indicating promising applications in solid state or molten salt batteries at low to intermediate temperatures.

## 1. Introduction

Na-β”-alumina (more commonly referred to as β”-alumina) is a sodium ion conductor, which is used as a solid electrolyte in sodium-sulfur batteries, sodium-nickel chloride batteries, and in alkali metal-based thermoelectric converters [1,2,3]. While significant attention to Na-β”-alumina dates back as early as in the 1960s when sodium-sulfur batteries were originally developed by Weber and Kummer at the Ford Motor Company [1], the relatively high working temperature in a molten sodium-sulfur battery has significantly limited their applications and development, and soon, they were almost completely replaced by more popular lithium ion batteries in mobile applications. However, with increasing demands on large-scale energy storage due to climate change and the bloom of a solid-state battery, applications of Na-β”-alumina are seemingly rising again because of the abundance of sodium as compared to lithium sources and the high ionic conductivity of about 1 S cm^−1^ in single crystal Na-β”-alumina and 0.2–0.4 S cm^−1^ in polycrystalline β”-alumina at 300 °C [4,5,6,7,8,9,10]. For example, recently, Fertig et al. have provided a detailed review on the potential revival of Na-β”-alumina for sodium solid-state batteries [4]. Ligon et al. have reported large planar Na-β”-alumina solid electrolytes (150 mm in diameter) for next generation Na-batteries [5]. Lee et al. have demonstrated a full-scale pilot model of a cost-effective sodium-nickel-iron chloride battery over 40 Ah using a Na-β”-alumina electrolyte [9]. Zhu et al. have reported that by ion exchange, a Na-β”-alumina-containing composite electrolyte may be ion exchanged with molten salts to lithium-ion or silver-ion conducting electrolytes [11], which may further expand the application of Na-β”-alumina in other types of batteries. 

The conventional process for the fabrication of dense samples of Na-β”-alumina consists of first calcining a mixture of Na_2_CO_3_, α-alumina, and LiNO_3_ (or MgO) at ~1250 °C in air, which leads to a powder mixture containing Na-β”-alumina, Na-β-alumina (β-alumina), and some NaAlO_2_. Powder compacts are then sintered in sealed platinum or MgO crucibles at ~1600 °C [12]. Sintering in sealed containers suppresses the loss of Na_2_O through the vapor phase. Densification occurs by a transient liquid phase mechanism. Sintered samples are subsequently heat-treated at a lower temperature (~1400 °C) to convert β-alumina into β”-alumina by reacting with NaAlO_2_. Usually, a small amount of NaAlO_2_ remains unreacted in β”-alumina as a thin film along grain boundaries, which makes it susceptible to degradation due to moisture in the atmosphere. Thus, Na-β”-alumina made by the conventional process is stored in desiccators. 

In concept, if an already-sintered α-alumina can be converted into Na-β”-alumina by reacting it with Na_2_O, it may be possible to avoid the formation of NaAlO_2_ along the grain boundaries, thus making it moisture-resistant. A possible vapor process for the conversion of sintered α-alumina into Na-β”-alumina involves exposing it to a vapor containing Na_2_O. The approximate composition of Na-β”-alumina is Na_2_O ~6Al_2_O_3_. For the conversion of α-alumina into Na-β”-alumina, the reaction is

Na_2_O + ~6Al_2_O_3_ → Na_2_O ~6Al_2_O_3_ (1)

**Figure A1:**
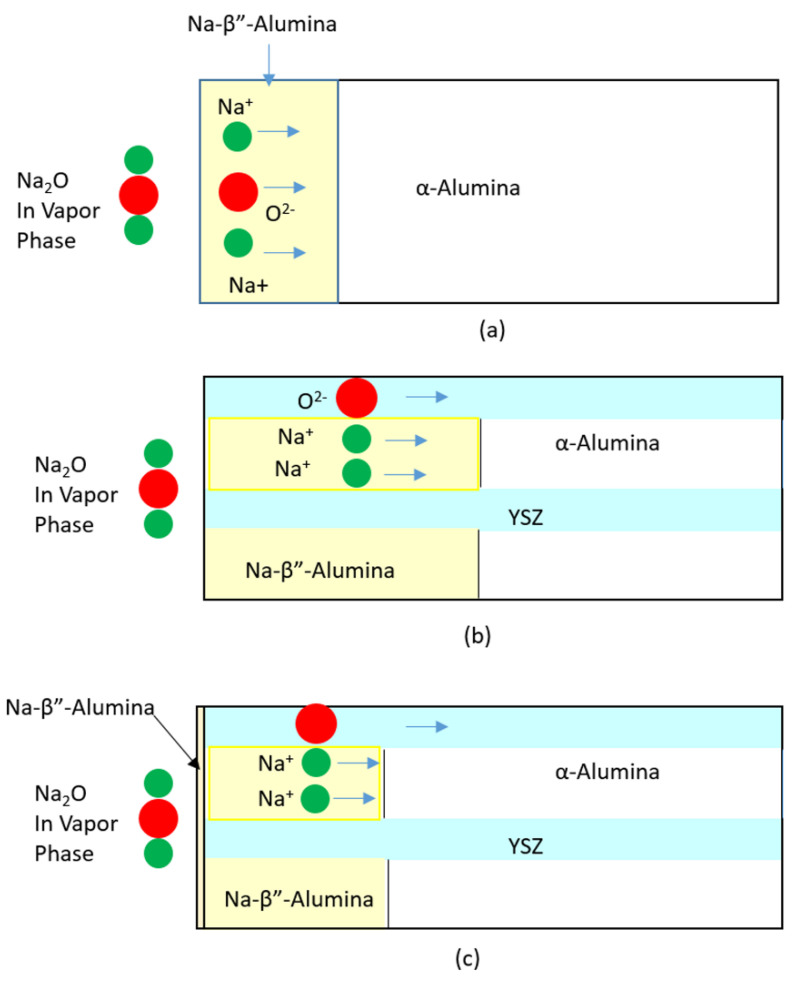
Schematics showing vapor phase conversion in (**a**) single phase α-alumina, (**b**) α-alumina + YSZ composite, and (**c**) α-alumina-coated α-alumina + YSZ composite. The schematics do not show grain structure in this one-dimensional model. The widths of YSZ, α-alumina, and Na-β”-alumina are essentially equal to the respective volume fractions.

If a fully dense sintered Al_2_O_3_ is exposed to Na_2_O vapor, a thin layer of Na-β”-alumina forms on the surface. Further conversion of the interior α-alumina requires the transport of Na_2_O (as coupled (ambipolar) transport of 2Na^+^ and O^2−^) through the formed Na-β”-alumina (Figure A1a in Appendix A). While the Na^+^ diffusivity through Na-β”-alumina is high, O^2−^ diffusivity is very low. Thus, the conversion kinetics are dictated by the diffusion coefficient of oxygen ions in Na-β”-alumina and are very sluggish. Furthermore, the conversion kinetics are diffusion-limited (parabolic). At 1300 °C, for example, the thickness of α-alumina converted into Na-β”-alumina in 16 h was only about 25 μm [13]. 

**Figure 1:**
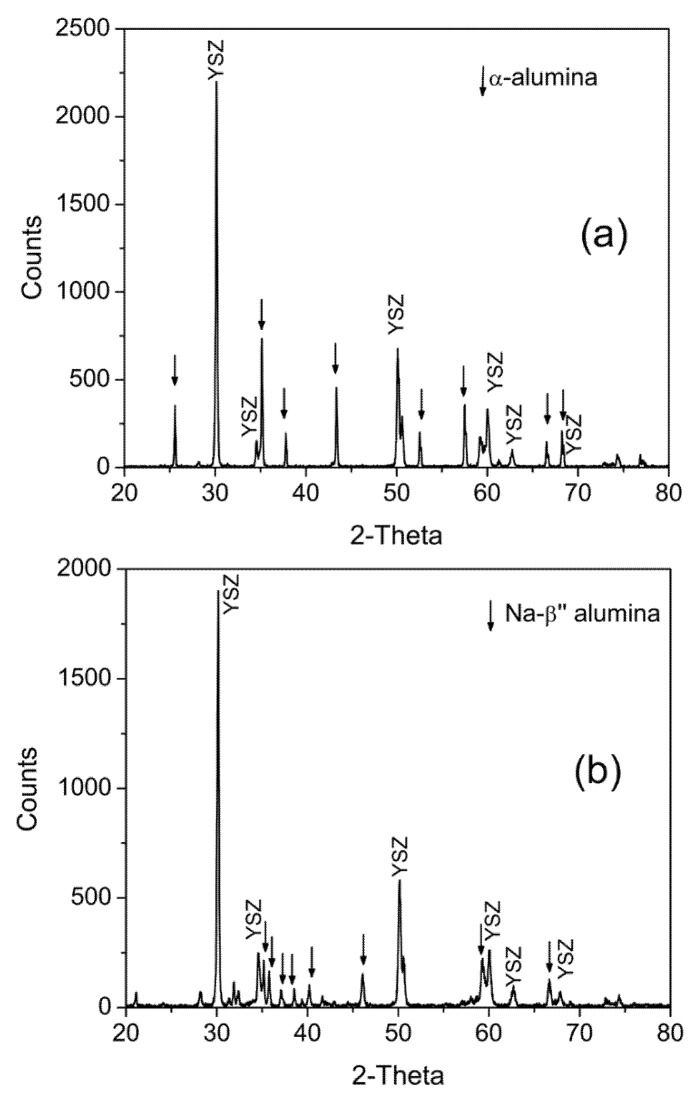
XRD patterns of α-Alumina + YSZ composites (**a**) before and (**b**) after subjecting to vapor phase conversion at 1250 °C.

In the novel vapor phase process, a two-phase composite of α-alumina and YSZ, with both phases being contiguous, is exposed to Na_2_O vapor [14]. Once α-alumina on the surface is converted to Na-β”-alumina, subsequent conversion of the interior α-alumina to Na-β”-alumina involves the transport of Na_2_O, such that 2Na^+^ transports through the formed Na-β”-alumina, while the O^2−^ transports through the YSZ phase (Figure 1b). More specifically, the effective diffusion of Na_2_O through the two phase mixture occurs by a coupled (ambipolar) transport of 2Na^+^ through Na-β”-alumina and O^2−^ through YSZ. Since the diffusion coefficient of O^2−^ through YSZ is much higher than that of O^2−^ through Na-β”-alumina, the kinetics of conversion of α-alumina + YSZ into Na-β”-alumina + YSZ is much faster than the conversion of single phase α-alumina into Na-β”-alumina. The resulting two-phase composite of Naβ”AY is also much stronger than conventional Na-β”-alumina and is moisture-resistant. 

Since the invention of the novel vapor phase process patented by Virkar et al. [14], it has been gaining attention in Na batteries [15,16,17,18,19]. A recent study has shown that a planar sodium nickel chloride battery demonstrated a specific energy density of as high as 350 Wh kg^−1^, operated at 190 °C over 1000 cycles. The composite electrolyte adopted in that battery consisted of α-alumina and 8YSZ, also with a volume ratio of 7:3 as starting materials, and then was converted via the vapor phase conversion process. Such a composite electrolyte also offered high mechanical strength. A flexural strength of higher than 300 MPa has been reported in literature [20,21], making them an excellent Na-conducting membrane in Na batteries.

The only available model reported so far on the kinetics of this novel vapor phase conversion process is the one reported by Parathasarathy and Virkar [22], where the kinetics of conversion of α-alumina + YSZ composites of various grain sizes over a range of temperatures between 1250 °C and 1400 °C were investigated. The experimental procedure involved packing sintered α-alumina + YSZ samples into Na-β”-alumina powder, heat treating at a given temperature for a period of time, cooling down to room temperature, and grinding/polishing the sample to measure (using a microscope) the conversion thickness x(t) as a function of cumulative heat treatment time t. The process thus is tedious, as it requires repeated grinding and polishing. Furthermore, this involves the destruction of the sample. Under some situations, where both conversion fraction and conductivity as a function of conversion time are of interest, the above thickness-based model may be insufficient, since it requires cutting of the sample. Therefore, an alternate model needs to be developed to fulfill the routine examination of this vapor phase process. 

The objective of the present work was to measure the sample weight and the external dimensions after various thermal treatments so that the kinetics of conversion could be measured more effectively without destroying the sample and, at the same time, minimizing the effort required in the actual measurements.

An equally important model was also developed and experimentally verified to measure the chemical diffusion coefficient of oxygen through single phase -β”-alumina by depositing a thin layer of α-alumina on the α-alumina + YSZ composite prior to conversion. The α-alumina-coated samples were subjected to the same conversion treatment as the uncoated samples. Both of the newly developed models may be used as effective tools for future studies and applications of the vapor phase process. Note that the concepts and models developed here may well fit to other binary or possibly ternary diffusion system where diffusion kinetics of certain mobile ions, such as oxygen-ion, proton, metal ions, etc., are of interest.

## 2. Materials and Methods

### 2.1. Preparation of α-Alumina + YSZ Samples

High purity α-alumina (AKP-53, Sumitomo Chemical) and 3YSZ (TZ-3Y, Tosoh Corporation, Tokyo, Japan) were mixed in a volume ratio of alumina:3YSZ of 7:3. A slurry of the powder mixture was made in distilled water with a small amount of ammonium poly methyl methacrylate (DARVAN C-N, R. T. Vanderbilt, Inc., New York, NY, USA), added as a dispersant and as a stabilizer. The slurry was planetary milled for 12 h, dried, and then heated to 500 °C for 5 h. The dried powder was sieved through a 70-mesh screen to remove any large agglomerates. A few grams of powder was placed in a circular die and uniaxially pressed under a force of 5 tons, followed by cold isostatic pressing at 30,000 psi. The disc was then pre-sintered at 1200 °C for 2 h and then polished on both sides to a finish of ~50 nm using an alumina suspension in water. After polishing, selected samples were either spin-coated or dip-coated with an alumina suspension depending on the desired thickness. After slowly heating the samples to 1000 °C to burnout the organic components, all of the samples were sintered in air at 1600 °C for 5 h. The weight and the dimensions (diameter and thickness) were measured as the baseline information. 

### 2.2. Formation of Na-β”-Alumina + YSZ Samples via Vapor Phase Conversion

Sintered discs were then buried in Na-β”-alumina powder (Materials and Systems Research, Inc., Salt Lake City, UT, USA) in an alumina crucible and covered with a lid. The crucible was placed in a furnace in air and heated to 1250 °C (5 °C min^−1^ ramp up rate), maintained for a period of time at temperature, and rapidly cooled to room temperature (>15 °C min^−1^ ramp down rate). The objective was to ensure that for most of the reaction time, the samples were at the heat treatment temperature. Since the process of vapor phase conversion of α-alumina + YSZ into Na-β”-alumina + YSZ is thermally activated, most of the conversion occurred isothermally at 1250 °C, and any conversion during heating to 1250 °C and cooling down from the heat treatment temperature could be neglected. This process was repeated several times until a maximum cumulative time of ~84 h. The dimensions of the samples and their weights were carefully recorded after each thermal treatment. For weight measurement, a balance with a resolution of 0.0001 g and repeatability <0.0002 g was used. The digital caliper used for dimension measurements had a resolution of 0.01 mm with negligible deviation in repeat measurements. In order to examine the conversion front at different conversion times, a small part of one of the samples was cut from the edge after each conversion period and fine polished for microstructure characterization. Samples used for weight measurements were not cut. 

### 2.3. Characterization

Similar samples were prepared for X-ray diffraction (XRD) and scanning electron microscopy (SEM). An Energy Dispersive Spectroscopy (EDS) elemental line scan was performed on a partially converted sample to examine the α-alumina + YSZ/Na-β”-alumina + YSZ interface. Density measurements were performed by the standard fluid immersion method on an as-sintered α-alumina + YSZ sample and a fully converted Naβ”AY sample. Conductivity was measured in air as a function of temperature using an AC Electrochemical Impedance Spectroscopy (EIS) with gold paste as electrodes. Measurements were conducted over a frequency range from 10 Hz to 1 MHz. The high frequency intercept was taken as the measure of conductivity.

## 3. Results and Discussion

### 3.1. X-ray-Diffraction

Figure 1 shows XRD patterns of an as-sintered α-alumina + YSZ sample and a fully converted Naβ”AY sample. In Figure 1a, all peaks are identified as belonging to either α-alumina or YSZ. Peaks belonging to α-alumina have been identified by arrows. In Figure 1b, which is for the converted sample, peaks belonging to Na-β”-alumina have been identified by arrows. No peaks belonging to α-alumina were observed in the converted sample. Some of the peaks belonging to Na-β-alumina overlapped with those of Na-β”-alumina. Thus, the presence of some Na-β-alumina cannot be ruled out. 

### 3.2. SEM and EDS Analysis

**Figure 2:**
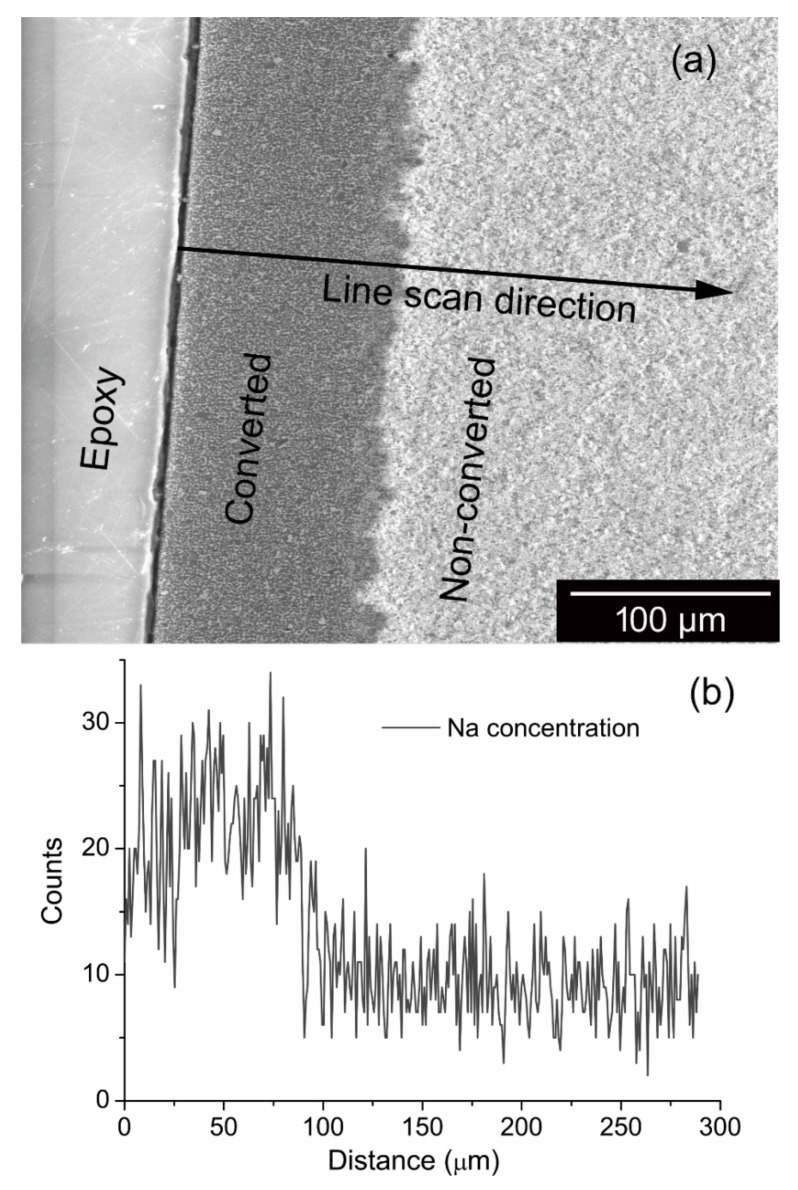
(**a**) An SEM image showing the converted and the pristine regions of the initially α-alumina + YSZ sample after 4 h of conversion at 1250 °C. (**b**) The corresponding EDS line scan is indicated by the arrow.

Figure 2a shows an SEM image of a polished section of a sample that had been heat-treated at 1250 °C for 4 h by packing in Na-β”-alumina powder. The converted region is darker in color. The average conversion thickness measured was ~110 μm. Figure 2b shows an EDS scan from the surface of the sample, across Na-β”-alumina + YSZ/α-alumina + YSZ interface and into the α-alumina + YSZ unconverted region. While the EDS scan was not smooth and showed considerable scatter, the demarcation between the converted region and the pristine region is clear. 

**Figure 3:**
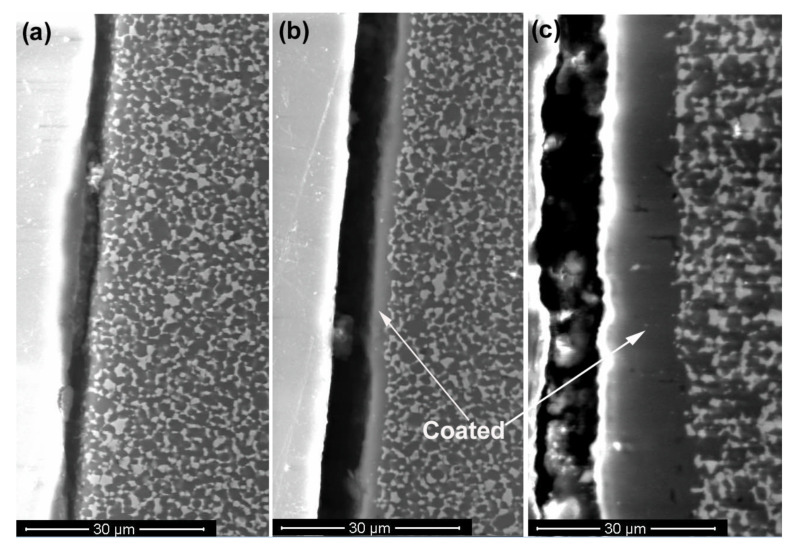
SEM images showing the enlarged view at the interface of (**a**) The uncoated region, (**b**) A ~2.5 µm Na-β”-alumina-coated sample, and (**c**) A ~15 µm Na-β”-alumina-coated sample.

Figure 3 shows SEM micrographs of the cross-sections of an uncoated sample, a sample with a ~2.5 μm-thick layer Na-β”-alumina (initially, an alumina layer of 2 μm-thickness) and a sample with a ~15 μm-thick layer of Na-β”-alumina (initially, an alumina layer of 12 μm-thickness) after conversion treatment for 4 h, 4 h, and 84 h, respectively. The micrographs in (b) and (c) thus correspond to samples that were initially coated with α-alumina. These layers fully converted to Na-β”-alumina. The interiors of all three samples contained Na-β”-alumina + YSZ. The dark phase is Na-β”-alumina; the light phase is YSZ. The Na-β”-alumina and YSZ phases were both contiguous, as would be required for coupled transport of Na_2_O to occur through the two phase mixture: Na^+^ through Na-β”-alumina and O^2−^ through YSZ [22].

**Figure 4:**
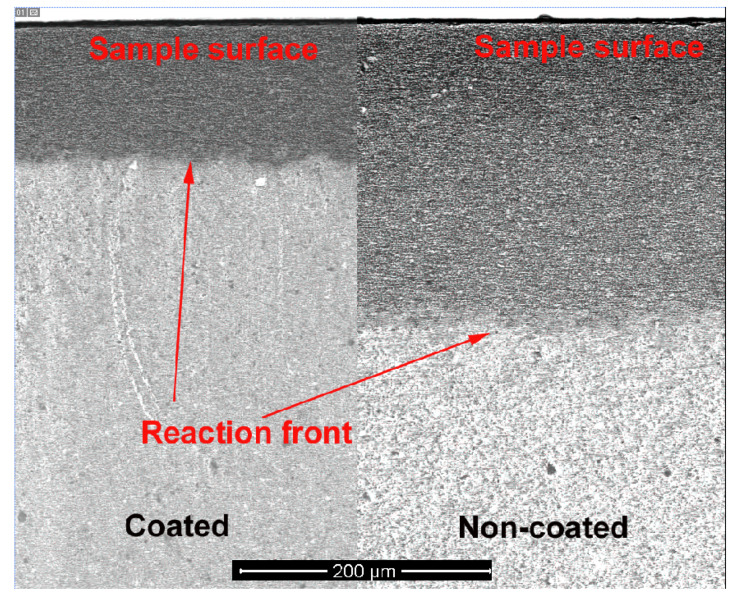
SEM images showing the conversion fronts for the uncoated and 2.5 µm Na-β”-alumina-coated regions after 6 h of conversion.

Figure 4 compares the conversion thicknesses of the alumina-coated (~2.5 μm Na-β”-alumina after conversion) portion of the sample, with part of the region that was uncoated, after conversion at 1250 °C for 6 h. The conversion thickness corresponding to the non-coated region was about twice that of the coated region. This shows that the presence of a 2.5-μm Na-β”-alumina surface coating substantially lowered the conversion kinetics. 

### 3.3. Estimation of the Kinetic Parameters on the Uncoated Samples

Appendix A gives the relevant equations describing the kinetics of conversion and the chemical diffusion coefficients. Equation (A1) gives the conversion thickness x corresponding to isothermal treatment time of t in terms of the effective diffusion coefficient, $D_{eff}$, and the effective interface transfer parameter, $k_{eff}$. Appendix B gives the kinetic equation in terms of the sample dimensions and the increase in weight as a function of time. The following describes an example of the estimation of the kinetic parameters on an uncoated sample. 

**Figure 5:**
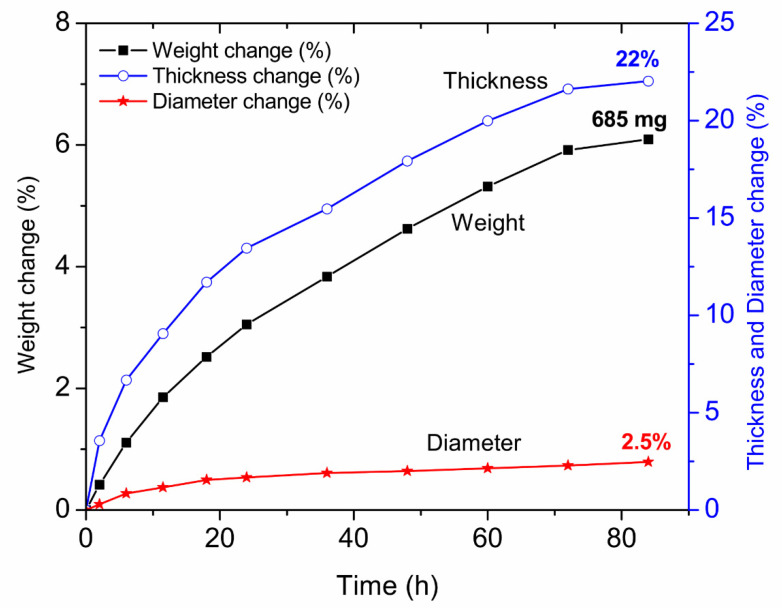
Measured weight and geometry change of an uncoated sample as a function of conversion time.

The density of the as-sintered α-alumina + YSZ samples, $\rho_{AY}$, was measured as 4.59 g cm^−3^. The initial thickness of the α-Al_2_O_3_ + YSZ disc sample was 3.64 mm, and the initial diameter was 29.54 mm. Figure 5 shows the measured weight, the thickness, and the diameter changes in percent with respect to the initial measurements. The initial weight of the sample, $m_{o}$, was 11.2452 g. After conversion for a cumulative time of 84 h at 1250 °C, the sample weight increased to 11.9302 g. That is, the sample weight increased by 685 mg after a cumulative conversion time of 84 h at 1250 °C. After 72 h, the weight increased only slightly. This indicated that the sample had fully or very close to fully converted after 84 h.

At 1250 °C. The sample cross-section confirmed that it had fully converted to Na-β”-alumina + YSZ. Thus, the final weight of the sample of 11.9302 g was taken as the weight after full conversion, that is, m(∞)= 11.9302 g. The density of the fully converted thinner sample prepared using the same procedure, $\rho_{\beta {}^{\prime \prime }Y}$, was measured as 3.94 g cm^−3^. From the measured values of $m_{o}$, m(∞), $\rho_{AY}$, and $\rho_{\beta {}^{\prime \prime }Y}$, the ratio $\frac{x{}^{\prime }(t)}{x(t)}$ corresponding to Equation (A16) was measured as

(2) $$\frac{x{}^{\prime }(t)}{x(t)}=\frac{l_{0}}{l(\infty )}=\frac{\rho_{AY}m_{o}}{\rho_{\beta {}^{\prime \prime }Y}m(\infty )}=0.81$$

The ratio

(3) $$\frac{m_{o}}{m(\infty )}=0.94$$

is used in Equation (A18).

The thickness measured after conversion (84 h) was 4.45 mm. Thus, $\frac{l_{o}}{l(\infty )}$ based on the thickness measurement is

(4) $$\frac{l_{o}}{l(\infty )}=0.82$$

The increase in thickness was ~22%. The corresponding increase in diameter was only ~2.5%. For this reason, the assumption that most of the dimensional change occurs along the thickness direction is reasonable. Based on the weight gain, it is concluded that the stoichiometry of Na-β”-alumina is almost exactly Na_2_O·6Al_2_O_3_. 

The kinetic equation in terms of weight change given in Equation (A19) is reproduced here

(5) $$\frac{{\left(\Delta m(t)\right)}^{2}}{4D_{eff}{\left(A\rho_{\beta {}^{\prime \prime }Y}\left(1-\frac{m_{o}}{m(\infty )}\right)\right)}^{2}}+\frac{\Delta m(t)}{2k_{eff}\left(A\rho_{\beta {}^{\prime \prime }Y}\left(1-\frac{m_{o}}{m(\infty )}\right)\right)}=t$$

The same equation can be written as

(6) $$\Delta m\left(t\right)=4D_{eff}{\left(A\rho_{\beta {}^{\prime \prime }Y}\left(1-\frac{m_{o}}{m(\infty )}\right)\right)}^{2}\frac{t}{\Delta m\left(t\right)}-\frac{2D_{eff}}{k_{eff}}\left(A\rho_{\beta {}^{\prime \prime }Y}\left(1-\frac{m_{o}}{m(\infty )}\right)\right)$$

**Figure 6:**
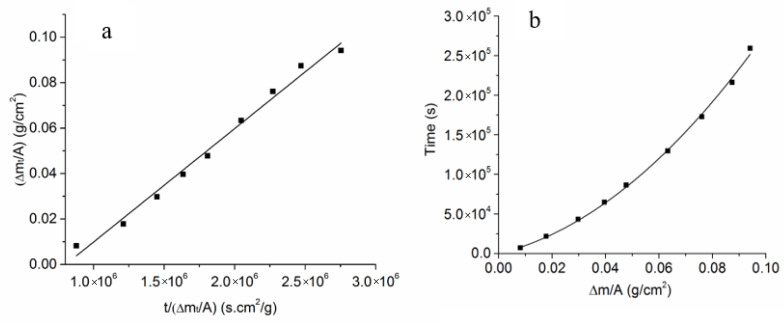
(**a**) A plot of $\frac{\Delta m\left(t\right)}{A}$ vs. $\frac{t}{\left(\frac{\Delta m\left(t\right)}{A}\right)}$ for the uncoated sample. From the slope and the intercept, both $D_{eff}$ and $k_{eff}$ can be determined. (**b**) A plot of t vs. $\frac{\Delta m\left(t\right)}{A}$ for the uncoated sample by a second order polynomial fitting with the intercept set as 0. From the 1st order and 2nd order coefficients, both $k_{eff}$ and $D_{eff}$ can be determined.

In Equations (5) and (6), the Δm(t) is in g. Equation (6) shows that a plot of Δm(t) vs. $\frac{t}{\Delta m\left(t\right)}$ should be linear, with the slope given by $4D_{eff}{\left(A\rho_{\beta {}^{\prime \prime }Y}\left(1-\frac{m_{o}}{m(\infty )}\right)\right)}^{2}$ and the intercept given by $-\frac{2D_{eff}}{k_{eff}}\left(A\rho_{\beta {}^{\prime \prime }Y}\left(1-\frac{m_{o}}{m(\infty )}\right)\right)$. Thus, from the slope and the intercept, one should be able to estimate $D_{eff}$ and $k_{eff}$. Figure 6a gives a plot of $\frac{\Delta m\left(t\right)}{A}$ vs. $\frac{t}{\left(\frac{\Delta m\left(t\right)}{A}\right)}$. Thus, the slope is $4D_{eff}{\left(\rho_{\beta {}^{\prime \prime }Y}\left(1-\frac{m_{o}}{m(\infty )}\right)\right)}^{2}$, and the intercept is $-\frac{2D_{eff}}{k_{eff}}\left(\rho_{\beta {}^{\prime \prime }Y}\left(1-\frac{m_{o}}{m(\infty )}\right)\right)$. As seen in Figure 6a, the plot is linear. The corresponding slope was 4.99 × 10^−8^ g^2^ cm^−4^ s^−1^, and the intercept was −0.04 g cm^−2^. It is significant that the intercept was negative, as required. The corresponding estimated values of the kinetic parameters are $D_{eff}≅1.74\times 10^{-7}$ cm^2^ s^−1^ and $k_{eff}≅2.33\times 10^{-6}$ cm s^−1^. The experimental data were directly fitted to Equation (5) by a polynomial fitting, as shown in Figure 6b. The estimated values were $D_{eff}≅1.76\times 10^{-7}$ cm^2^ s^−1^ and $k_{eff}≅2.29\times 10^{-6}$ cm s^−1^, which were in good agreement with the results of the linear plot in Figure 6a. The estimated value of $D_{eff}$ was in good agreement with that measured by Parthasarrathy and Virkar [22] based on conversion thickness, which was $D_{eff}≅1.5\times 10^{-7}$ cm^2^ s^−1^. The $k_{eff}$ measured by Parthasarathy and Virkar [22] ranged between ~3.6 × 10^−7^ cm s^−1^ for large-grained samples to ~1.0 × 10^−6^ cm s^−1^ for fine-grained samples. The $k_{eff}$ estimated in the present work was thus between 3- and 10-times larger than in the study by Parthasarathy and Virkar. Given the possible differences in microstructures, the agreement is deemed reasonable. The present work thus shows that weight measurements can be used to estimate both kinetic parameters without having to section and polish after each thermal treatment.

### 3.4. Effects of Na-β”-Alumina Coating on Conversion Kinetics

**Figure 7:**
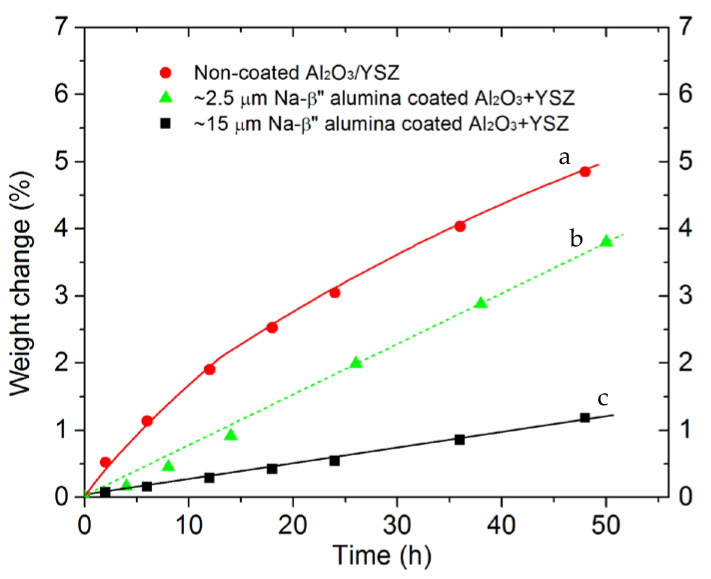
Comparison in weight change for (**a**) uncoated, (**b**) ~2.5 µm Na-β”-alumina-coated, and (**c**) ~15 µm Na-β”-alumina-coated samples as a function of conversion time at 1250 °C.

Figure 7 compares the measured weight changes for the uncoated, 2.5 µm coated, and 15 µm coated (thicknesses correspond to the formed Na-β”-alumina after conversion) disc samples. For the uncoated and 15 μm coated samples, the figure shows the actual measured weight changes (in percent) as a function of time. For the sample with a 2.5 μm coating, the actual conversion thickness was measured as a function of time. In order to graph the data on the same plot, the expected weight changes in the 2.5 µm coated sample were calculated from the measured conversion thickness using Equation (A15). In Figure 7, these data are shown by a dashed line. The uncoated sample showed a much higher weight percentage change than the coated samples. As seen in the figure, the uncoated sample exhibited the largest weight gain (faster kinetics), and the 15 μm coated sample showed the lowest (slowest kinetics of the three samples tested). Also significant is the observation that over the duration of the tests, the weight gain vs. time plots were linear for both of the coated samples, indicating that the kinetics can be described as interface-controlled. 

### 3.5. Estimation of the Kinetic Parameter for the Coated Samples

**Figure 8:**
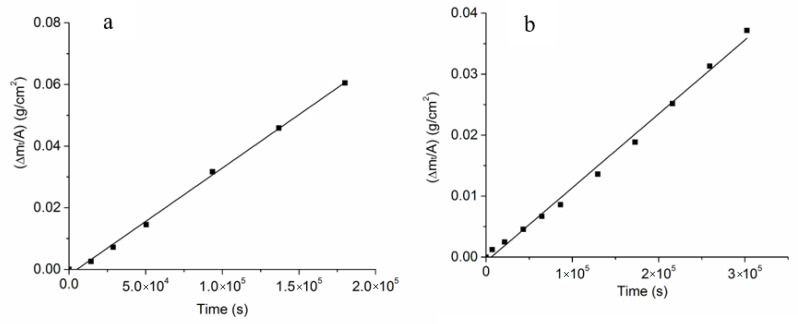
(**a**) A plot of $\frac{\Delta m\left(t\right)}{A}$ vs. t for a sample with a 2.5 μm Na-β”-alumina surface coating. From the slope, the $k_{eff}$ is obtained. (**b**) A plot of $\frac{\Delta m\left(t\right)}{A}$ vs. t for a sample with a 15 μm Na-β”-alumina surface coating. From the slope, the $k_{eff}$ is obtained.

Figure 8a,b show plots of $\frac{\Delta m_{t}}{A}$ vs. t for the two coated samples. Note that these plots are linear, indicating that the kinetics is interface-controlled. Equation (A19) then becomes

(7) $$\frac{\Delta m\left(t\right)}{A}≅2k_{eff}\left(\rho_{\beta {}^{\prime \prime }Y}\left(1-\frac{m_{o}}{m\left(\infty \right)}\right)\right)t$$

Thus, the slope is given by

(8) $$2k_{eff}\left(\rho_{\beta {}^{\prime \prime }Y}\left(1-\frac{m_{o}}{m\left(\infty \right)}\right)\right)$$

from which the $k_{eff}$ can be estimated. The estimated values of $k_{eff}$ were 6.45 × 10^−7^ cm s^−1^ for the sample with a 2.5 μm Na-β”-alumina coating and 2.26 × 10^−7^ cm s^−1^ for the sample with a 15 μm Na-β”-alumina coating. This $k_{eff}$ has a number of series contributions, as discussed below. 

**Figure 9:**
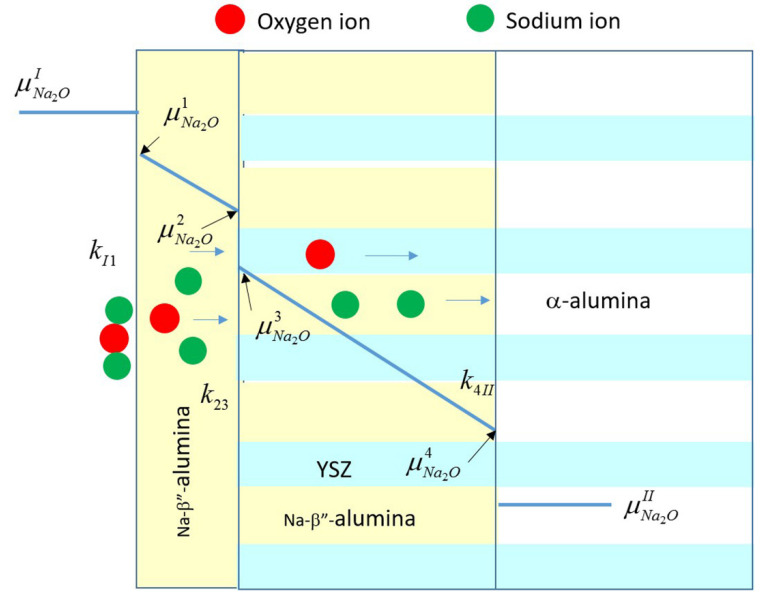
A schematic showing the variation of the chemical potential of Na_2_O, $\mu_{Na_{2}O}$, through a sample with surface layer of Na-β”-alumina of thickness, δ.

Figure 9 shows a schematic variation of the chemical potential of Na_2_O from the gas phase, $\mu_{Na_{2}O}^{I}$; just inside the single phase Na-β”-alumina close to the gas phase, $\mu_{Na_{2}O}^{1}$; in the single phase Na-β”-alumina close to the single phase Na-β”-alumina/Na-β”-alumina + YSZ interface, $\mu_{Na_{2}O}^{2}$; just inside the Na-β”-alumina + YSZ close to the single phase Na-β”-alumina/Na-β”-alumina + YSZ interface, $\mu_{Na_{2}O}^{3}$; inside the Na-β”-alumina + YSZ close to the Na-β”-alumina + YSZ/α-alumina + YSZ interface, $\mu_{Na_{2}O}^{4}$; and inside the α-alumina + YSZ close to the Na-β”-alumina + YSZ/α-alumina + YSZ interface, $\mu_{Na_{2}O}^{II}$. The transfer of Na_2_O across the three interfaces can be described by three interface kinetic parameters, namely, $k_{I1}$, $k_{23}$, and $k_{4II}$. The transport of Na_2_O through single-phase Na-β”-alumina is dictated by the chemical diffusion coefficient of Na_2_O, ${\tilde{D}}_{Na_{2}O}^{\beta {}^{\prime \prime }}$. However, this transport occurs through a layer of fixed thickness, δ. Thus, it reflects as an interface step, with the corresponding $k_{12}$ given by $\frac{{\tilde{D}}_{Na_{2}O}^{\beta {}^{\prime \prime }}}{\delta }$. The measured interface parameter, $k_{eff}$, is thus related to these parameters by the following equation.

(9) $$\frac{1}{k_{eff}}=\frac{1}{k_{I1}}+\frac{1}{k_{23}}+\frac{1}{k_{4II}}+\frac{\delta }{{\tilde{D}}_{Na_{2}O}^{\beta {}^{\prime \prime }}}$$

**Figure 10:**
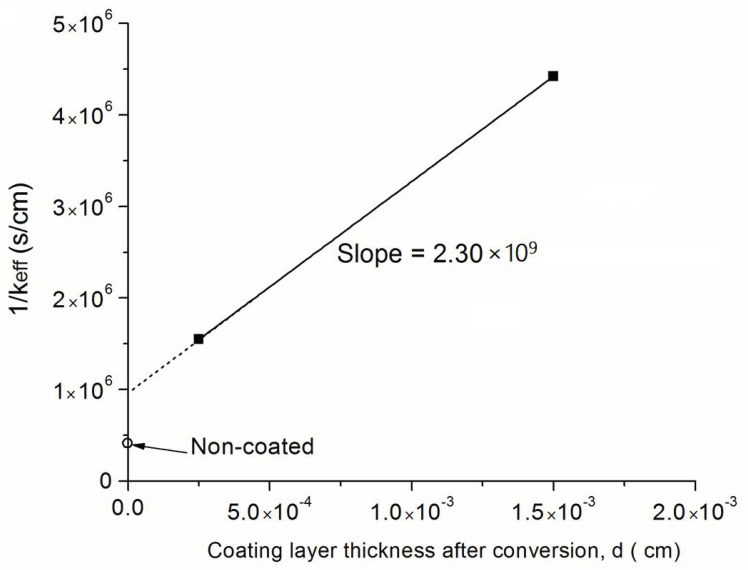
A plot of $1/k_{eff}$ vs. the Naβ”-alumina layer thickness, δ. Also shown in the figure is the $k_{eff}$ for the uncoated sample.

The preceding suggests that a plot of $\frac{1}{k_{eff}}$ vs. layer thickness, δ, should be a straight line with the slope given by $\frac{1}{{\tilde{D}}_{Na_{2}O}^{\beta {}^{\prime \prime }}}$ and the intercept given by $\frac{1}{k_{I1}}+\frac{1}{k_{23}}+\frac{1}{k_{4II}}$. Figure 10 is a plot of $\frac{1}{k_{eff}}$ vs. δ. In this study, measurements were conducted on samples with only two different thicknesses. Thus, there are only two data points. The straight line shown is thus through these two points. Furthermore, plotted on the same plot, however, is $\frac{1}{k_{eff}}$ for the uncoated sample, for which δ=0. For the uncoated sample, the relevant equation is

(10) $$\frac{1}{k_{eff}}=\frac{1}{k_{I3}}+\frac{1}{k_{4II}}$$

where $k_{I3}$ corresponds to the interface transfer parameter at the gas phase/Na-β”-alumina + YSZ interface. 

Note that

(11) $$\frac{1}{k_{I3}}+\frac{1}{k_{4II}}\neq \frac{1}{k_{I1}}+\frac{1}{k_{23}}+\frac{1}{k_{4II}}$$

From Figure 10, it is observed that the

(12) $$\frac{1}{k_{I3}}+\frac{1}{k_{4II}}<\frac{1}{k_{I1}}+\frac{1}{k_{23}}+\frac{1}{k_{4II}}$$

or

(13) $$\frac{1}{k_{I3}}<\frac{1}{k_{I1}}+\frac{1}{k_{23}}$$

or

(14) $$k_{I3}>\frac{k_{I1}k_{23}}{k_{I1}+k_{23}}$$

At this stage, it is not possible to separately determine the different interface transfer parameters. 

From the slope, the chemical diffusion coefficient of Na_2_O through single phase Na-β”-alumina is given by ${\tilde{D}}_{Na_{2}O}^{\beta {}^{\prime \prime }}≅4.35\times 10^{-10}$ cm^2^ s^−1^. By contrast, the $D_{eff}$ determined for coupled transport of Na_2_O as 2Na^+^ through Na-β”-alumina and O^2−^ through YSZ was ~1.74 × 10^−7^ cm^2^ s^−1^. That is, ${\tilde{D}}_{Na_{2}O}^{\beta {}^{\prime \prime }/YSZ}$ was over 400 times larger than ${\tilde{D}}_{Na_{2}O}^{\beta {}^{\prime \prime }}$. As given in Equation (A6), $D_{eff}$ for the two-phase sample is given by

(15) $$D_{eff}={\tilde{D}}_{Na_{2}O}^{\beta {}^{\prime \prime }/YSZ}\frac{2V_{m}}{f}\Delta C_{Na_{2}O}^{\beta {}^{\prime \prime }}$$

wherein

(16) $${\tilde{D}}_{Na_{2}O}^{\beta {}^{\prime \prime }/YSZ}=\frac{C_{O^{2-}}^{YSZ}D_{O^{2-}}^{YSZ}V_{YSZ}}{C_{Na_{2}O}^{\beta {}^{\prime \prime }}}\frac{1}{k_{B}T}\left(\frac{\partial \mu_{Na_{2}O}}{\partial \ln C_{Na_{2}O}^{\beta {}^{\prime \prime }}}\right)$$

That is, the ${\tilde{D}}_{Na_{2}O}^{\beta {}^{\prime \prime }/YSZ}$ is proportional to the diffusion coefficient of O^2−^ in YSZ, namely, $D_{O^{2-}}^{YSZ}$. However, in single-phase Na-β”-alumina, the Na_2_O chemical diffusion coefficient is given by

(17) $${\tilde{D}}_{Na_{2}O}^{\beta {}^{\prime \prime }}=C_{O^{2-}}^{\beta {}^{\prime \prime }}D_{O^{2-}}^{\beta {}^{\prime \prime }}\frac{1}{k_{B}T}\left(\frac{\partial \mu_{Na_{2}O}^{\beta {}^{\prime \prime }}}{\partial C_{Na_{2}O}^{\beta {}^{\prime \prime }}}\right)$$

That is, the ${\tilde{D}}_{Na_{2}O}^{\beta {}^{\prime \prime }}$ is proportional to the diffusion coefficient of O^2−^ in Na-β”-alumina. It is well known that oxygen diffusion is much faster in YSZ, an oxygen ion conductor, than in Na-β”-alumina. The present results are consistent with this expectation. Table 1 lists the measured $k_{eff}$ and $D_{eff}$ for alumina-coated and non-coated samples.

### 3.6. Measurement of Ionic Conductivity

**Figure 11:**
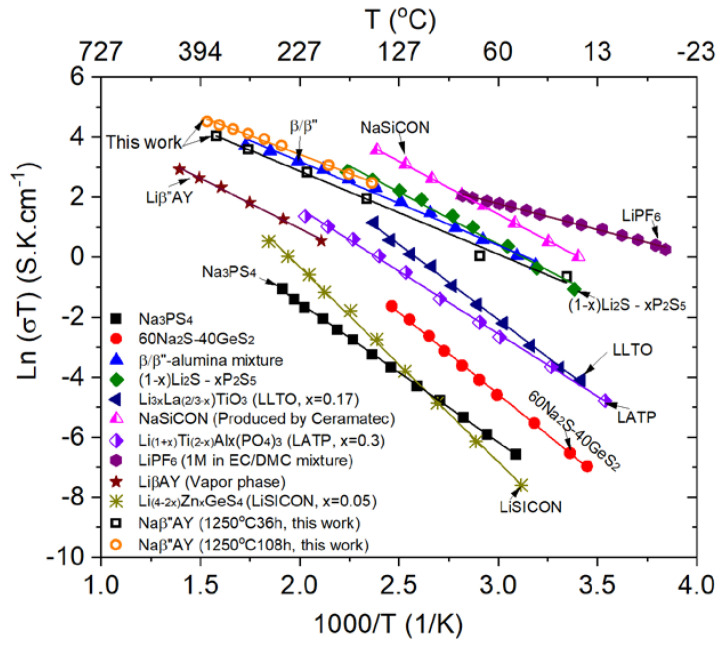
An Arrhenius plot of the measured conductivity of NaβAY and some common Na and Li electrolytes. Conductivity sources: Na_3_PS_4_ [23], 60Na_2_S-40GeS_2_ [23], β/β”-alumina mixture [15], (1 − x)Li_2_S − xP_2_S_5_ [23], Li_3x_La_(2/3−x)_TiO_3_ (LLTO, x = 0.17) [27], NaSiCON (Produced by Ceramatec) [23], Li_(1+x)_Ti_(2−x)_Al_x_(PO_4_)_3_ (LATP, x = 0.3 [29], LiPF_6_ (1M in EC/DMC mixture) [26], Liβ”AY (Vapor phase) [25], Li_(4−2x)_Zn_x_GeS_4_ (LiSICON, x = 0.05) [28].

Conductivity was measured on a disc sample of 0.9-mm-thickness that had been converted at 1250 °C for 36 h and a disc sample of 3.7-mm-thickness that had been converted at 1250 °C for 108 h. The heat treatment was long enough to ensure that the entire samples converted into Naβ”AY. Figure 11 shows an Arrhenius plots for the Naβ”AY composite and along with some common Na and Li electrolytes for better comparison [23,24,25,26,27,28,29]. Over the range of temperatures measured, the oxygen ion conductivity of YSZ was much lower than the sodium ion conductivity of Na-β”-alumina. Thus, the measured conductivity is attributed exclusively to sodium ion conduction. The measured activation energies of ~23.2 kJ mol^−1^ (after 36 h conversion) and ~21.2 k J mol^−1^ (after 108 h conversion) were also in accordance with the previously reported activation energy of Na^+^-ion conduction, especially in fine-grained Na-β”-alumina [10]. At 300 °C, the resistivity was measured as ~16 Ω cm (conductivity of 6.3 × 10^−2^ S cm^−1^) and ~8 Ω cm (conductivity of 1.3 × 10^−1^ S cm^−1^) for the samples converted for 36 h and 108 h, respectively. The calculated conductivity based on the best linear fitting of the Arrhenius plot at 25 °C was 1.4 × 10^−3^ and 1.6 × 10^−3^ S cm^−1^ for the samples converted for 36 h and 108 h, respectively. 

An interesting feature revealed by this combined plot is that the activation energies of the listed electrolytes do not change vastly regardless of the crystal structure or conduction mechanisms. In comparison with a liquid electrolyte, most of the solid electrolytes show better conductivity at a higher temperature regime. The comparison in the solid electrolytes indicates that the Naβ”AY composite electrolyte showed higher conductivity than most of the listed electrolytes, other than the mixed β/β”-alumina electrolyte and the NaSICON-type Na electrolyte. However, when mechanical strength is of a concern, Naβ”AY (~300 MPa flexural strength [20,21]) may be a better choice, due to a higher mechanical strength than that of NaSICON electrolyte, with ~100 MPa flexural strength [30]. Therefore, the Na”βAY composite produced by the vapor phase conversion process seems a promising solid electrolyte for Na battery applications. It is to be noted that although the Li-β”-alumina + YSZ electrolyte (Liβ”AY) synthesized by the vapor phase conversion process showed lower conductivity as compared with the Naβ”AY electrolyte, its conductivity was still higher than a few of the listed Li solid electrolytes.

## 4. Conclusions

The vapor phase conversion of α-alumina + YSZ into Na-β’’ alumina + YSZ (Naβ”AY) was investigated using the measurement of weight gain and external sample dimensions as a function of time. Vapor phase conversion studies were also conducted on samples coated with thin layers of α-alumina on the one surface, which during the process, first converted into a layer of Na-β”-alumina. In the coated samples, the transport of Na_2_O occurred through single phase Na-β”-alumina prior to the conversion of the interior α-alumina in the α-alumina + YSZ composite. From the measurement of conversion kinetics of the uncoated sample, the effective diffusion coefficient, $D_{eff}$, and effective interface transfer parameter, $k_{eff}$, were determined. At 1250 °C, the measured $D_{eff}$ was 1.74 × 10^−7^ cm^2^ s^−1^, in agreement with the previously reported results of Parthasarathy and Virkar [22]. The measured $k_{eff}$ was 2.33 × 10^−6^ cm s^−1^, which is within an order of magnitude of the values reported by Parthasarathy and Virkar [22]. Some difference may be related to the possible differences in microstructural details. The $D_{eff}$ is a measure of the chemical diffusion coefficient of Na_2_O in the two-phase Naβ”AY composite, ${\tilde{D}}_{Na_{2}O}^{\beta {}^{\prime \prime }/YSZ}$, which is proportional to the oxygen ion diffusion in YSZ. 

The measured kinetics of conversion of the coated samples were found to be linear in time, indicating interface-controlled kinetics. Since the chemical diffusion of Na_2_O must first occur through the surface Na-β”-alumina layer, it reflects as an interface step which is proportional to the oxygen diffusion coefficient through single-phase Na-β”-alumina. From the dependence of conversion kinetics on the thickness of the surface Na-β”-alumina layer, the chemical diffusion coefficient of Na_2_O through Na-β”-alumina, ${\tilde{D}}_{Na_{2}O}^{\beta {}^{\prime \prime }}$, was estimated. The estimated value was 4.35 × 10^−10^ cm^2^ s^−1^, which is over 400-times smaller than ${\tilde{D}}_{Na_{2}O}^{\beta {}^{\prime \prime }/YSZ}$. The measured ${\tilde{D}}_{Na_{2}O}^{\beta {}^{\prime \prime }}$ was proportional to the oxygen diffusion coefficient through Na-β”-alumina. The present work thus provides a method to determine the chemical diffusion coefficient of Na_2_O through a Naβ”AY composite, as well as through single-phase Na-β”-alumina. This method may have applicability to other systems. 

The measured highest conductivity of the converted Naβ”AY was 1.6 × 10^−3^ S cm^−1^ at 25 °C and 1.3 × 10^−1^ S cm^−1^ at 300 °C. These values are higher than many single-phase Li or Na solid electrolytes, indicating the Naβ”AY composite synthesized by the vapor phase conversion process may be used as a promising electrolyte membrane for Na batteries. 

## Figures and Tables

**Table 1:** Summary of measured $k_{eff}$ and $D_{eff}$ for alumina-coated and non-coated samples.

Samples	$k_{eff}\;(\mathrm{cm}\;s^{-1})$	$D_{eff}\;({\mathrm{cm}}^{2}\;s^{-1})$
Non-coated α-alumina/YSZ composite	2.33 × 10^−6^	1.74 × 10^−7^
~2.5 µm Na-β”-alumina-coated α-alumina + YSZ composite	6.45 × 10^−7^	1.74 × 10^−7^
~15 µm Na-β”-alumina-coated α-alumina + YSZ composite	2.26 × 10^−7^	1.74 × 10^−7^

**Figure A2:**
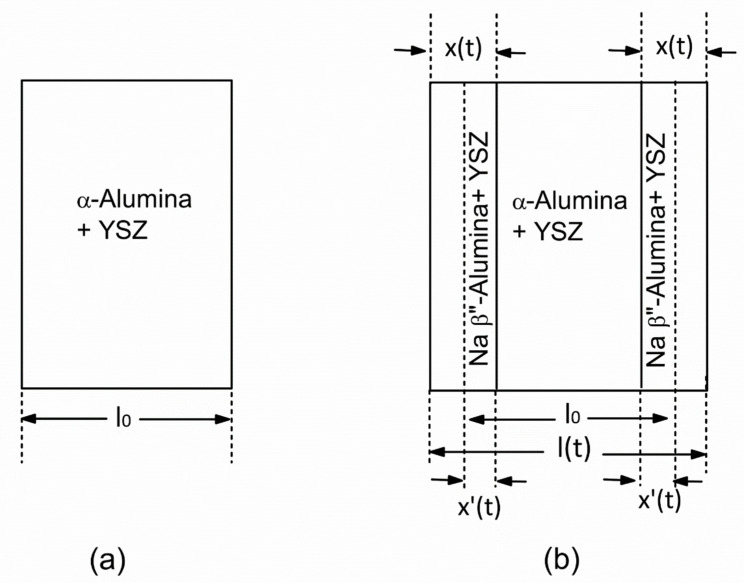
Schematics showing (**a**) a sintered α-alumina + YSZ sample before conversion and (**b**) after partial conversion. $x{}^{\prime }(t)$ is the initial thickness of α-alumina + YSZ which converted into Na-β”-alumina + YSZ of thickness x(t), in a conversion time of t.

## Data Availability

The data that support the plots within this paper are available from the corresponding authors upon reasonable request.

## Appendix A. Vapor Phase Conversion Kinetics and Transport Parameters

The vapor phase conversion of α-Alumina + YSZ (or with a thin α-alumina coating layer) to Na-β’’-alumina/YSZ consists of three steps in series. (a) Step 1: Na_2_O from the vapor phase is adsorbed on the Na-β’’ alumina + YSZ (or pure Na-β’’-alumina for an alumina-coated sample) surface. (b) Step 2: coupled diffusion of 2Na^+^ and O^2−^ through converted Na-β’’-alumina + YSZ in the composite or through, first, a thin layer of Na-β’’ alumina single phase, followed by coupled diffusion through a Na-β’’ alumina + YSZ composite for the coated sample. (c) Step 3: conversion of α-Alumina + YSZ to Na-β’’ alumina + YSZ composite at the reaction front; this requires lateral transport of O^2−^ ions arriving from the YSZ phase through Na-β’’-alumina, α-Alumina, or along the α-Alumina/Na-β’’-alumina interface, as discussed by Parthasarathy and Virkar [22]. Since in both coated and non-coated samples, the reaction front is the same, the interfacial kinetic parameter at this interface should be the same. This transport at the reaction front is similar to the conversion of γ-Fe into pearlite (α-Fe + Fe_3_C) in low carbon steels [31].

The kinetics of the conversion process of α-alumina + YSZ into Na-β”-alumina + YSZ was modeled by Parathasarthy and Virkar [22]. Figure A1b shows a schematic. Isothermal conversion kinetics could be described by an equation of the form

(A1) $$\frac{x^{2}}{D_{eff}}+\frac{x}{k_{eff}}=t$$

where x is the thickness converted in time t, $D_{eff}$ is the effective diffusion coefficient of Na_2_O for ambipolar transport of Na_2_O through the composite with 2Na^+^ transporting through Na-β”-alumina and O^2−^ transporting through the YSZ, and $k_{eff}$ is the interface transfer parameter that includes processes at the vapor phase/Na-β”-alumina + YSZ interface and also at the Na-β”-alumina + YSZ/α-alumina + YSZ reaction front. The $D_{eff}$ is given by

(A2) $$D_{eff}=\frac{2V_{YSZ}C_{O^{2-}}^{YSZ}D_{O^{2-}}^{YSZ}\left(\mu_{Na_{2}O}^{I}-\mu_{Na_{2}O}^{II}\right)V_{m}}{k_{B}Tf}$$

where $V_{m}$ is the molar volume of Na-β”-alumina, $\mu_{Na_{2}O}^{I}$ is the chemical potential of Na_2_O in gas phase at the Na_2_O-gas phase/Na-β”-alumina interface, $\mu_{Na_{2}O}^{II}$ is the chemical potential of Na_2_O in α-alumina at the Na-β”-alumina + YSZ/α-alumina + YSZ interface, and f is a factor which includes the dimensional change that occurs when α-alumina in the composite converts into Na-β”-alumina. We will write Equation (A2) as

(A3) $$D_{eff}=\frac{2V_{YSZ}C_{O^{2-}}^{YSZ}D_{O^{2-}}^{YSZ}\left(\mu_{Na_{2}O}^{I}-\mu_{Na_{2}O}^{II}\right)\left(C_{Na_{2}O}^{\beta {}^{\prime \prime }I}-C_{Na_{2}O}^{\beta {}^{\prime \prime }II}\right)V_{m}}{k_{B}T\left(C_{Na_{2}O}^{\beta {}^{\prime \prime }I}-C_{Na_{2}O}^{\beta {}^{\prime \prime }II}\right)f}$$

or

(A4) $$D_{eff}=\frac{2V_{YSZ}C_{O^{2-}}^{YSZ}D_{O^{2-}}^{YSZ}\left(C_{Na_{2}O}^{\beta {}^{\prime \prime }I}-C_{Na_{2}O}^{\beta {}^{\prime \prime }II}\right)V_{m}}{k_{B}Tf}\left(\frac{\partial \mu_{Na_{2}O}^{\beta {}^{\prime \prime }}}{\partial C_{Na_{2}O}^{\beta {}^{\prime \prime }}}\right)$$

or

(A5) $$D_{eff}=\frac{2V_{YSZ}C_{O^{2-}}^{YSZ}D_{O^{2-}}^{YSZ}}{k_{B}T}\left(\frac{\partial \mu_{Na_{2}O}^{\beta {}^{\prime \prime }}}{\partial C_{Na_{2}O}^{\beta {}^{\prime \prime }}}\right)\frac{V_{m}}{f}\Delta C_{Na_{2}O}^{\beta {}^{\prime \prime }}$$

where $\Delta C_{Na_{2}O}^{\beta {}^{\prime \prime }}$ denotes the difference in Na_2_O concentration in Na-β”-alumina from the Na_2_O-gas phase/Na-β”-alumina boundary to the Na-β”-alumina/α-alumina boundary. A comparison of Equations (A2) and (A5) shows that

(A6) $$D_{eff}={\tilde{D}}_{Na_{2}O}^{\beta {}^{\prime \prime }/YSZ}\frac{2V_{m}}{f}\Delta C_{Na_{2}O}^{\beta {}^{\prime \prime }}$$

Thus, the estimated $D_{eff}$ measured from conversion kinetics is a measure of the chemical diffusion coefficient of Na_2_O through the two-phase Na-β”-alumina + YSZ composite, namely, ${\tilde{D}}_{Na_{2}O}^{\beta {}^{\prime \prime }/YSZ}$, since $\frac{2V_{m}}{f}\Delta C_{Na_{2}O}^{\beta {}^{\prime \prime }}$ is a dimensionless constant. This chemical diffusion coefficient is proportional to the O^2−^ diffusion coefficient through YSZ.

The $k_{eff}$ also depends on the chemical potentials of Na_2_O. Equation (A1) assumes that the conversion thickness at the beginning of the isothermal process is negligible.

Figure A1a shows a schematic of the conversion of single phase α-alumina into Na-β”-alumina by the vapor phase process. In this case, the effective diffusion coefficient, as determined from conversion kinetics, would given by

(A7) $$D_{eff}={\tilde{D}}_{Na_{2}O}^{\beta {}^{\prime \prime }}\frac{2V_{m}}{f{}^{\prime }}\Delta C_{Na_{2}O}^{\beta {}^{\prime \prime }}$$

where $f{}^{\prime }$ is the factor that includes the dimensional change when α-alumina converts into Na-β”-alumina. As given in Equation (A7), the effective diffusion coefficient is proportional to the chemical diffusion coefficient of Na_2_O through Na-β”-alumina, which is effectively proportional to the O^2−^ diffusion coefficient through single phase Na-β”-alumina. This is expected to be very low compared to the O^2−^ diffusion coefficient through YSZ. Thus, we expect that

(A8) $${\tilde{D}}_{Na_{2}O}^{\beta {}^{\prime \prime }/YSZ}>>{\tilde{D}}_{Na_{2}O}^{\beta {}^{\prime \prime }}$$

Figure A1c schematically shows the conversion process in thin α-alumina-coated α-alumina + YSZ composites. Initially, the thin α-alumina layer is converted into Na-β”-alumina, the kinetics of which is governed by the effective diffusion coefficient, corresponding to transport through single-phase Na-β”-alumina, which is governed by oxygen diffusion through single-phase Na-β”-alumina. Once this layer is fully converted into Na-β”-alumina, the conversion of α-alumina in the interior α-alumina + YSZ composite begins. This conversion of the interior α-alumina into Na-β”-alumina occurs by coupled transport of 2Na^+^ through Na-β”-alumina and of O^2−^ through YSZ. However, this conversion requires a continual supply of Na_2_O, which must transport through the thin surface layer of the single-phase Na-β”-alumina of a fixed thickness. Because of the fixed thickness of this layer, the overall conversion kinetics contain an additional ‘interface’ transfer step, corresponding to Na_2_O diffusion through the single-phase Na-β”-alumina of a fixed thickness, δ. That is, the transport of Na_2_O through the single-phase Na-β”-alumina layer is equivalent to an additional interface step given by

(A9) $$k_{\beta {}^{\prime \prime }}\propto \frac{D_{O^{2-}}^{\beta {}^{\prime \prime }}}{\delta }$$

The larger the layer thickness, the smaller is the $k_{\beta {}^{\prime \prime }}$, and the slower is the kinetics. This suggests that the kinetics of conversion should be slower in samples coated with a Na-β”-alumina layer and that by comparing the kinetics of conversion of samples initially coated with two different thicknesses of alumina layers, it should be possible to estimate the diffusion coefficient of O^2-^ through a single phase Na-β”-alumina.

## Appendix B. Study of Kinetics of Vapor Phase Conversion by Weight Measurement

Figure A2 shows a schematic of the conversion process of an α-alumina + YSZ composite. The following analysis assumes that the samples are in the form of thin discs. When α-alumina is converted into Na-β”-alumina, there is both an increase in weight, since Na_2_O reacts with Al_2_O_3_ to form Na-β”-alumina (Na_2_O~6Al_2_O_3_), and an increase in volume, since the density of Na-β”-alumina is lower than that of Al_2_O_3_, and it also includes Na_2_O, which is an additional reason for the increase in volume. Prior work has shown that if the disks are sufficiently thin, most of the dimensional change occurs in the thin direction, and the disk diameter remains essentially unchanged. If the initial sample thickness is $l_{o}$, the cross-sectional area is A; then, the initial sample volume is $V_{o}=Al_{o}$. The density of the α-alumina + YSZ composite is $\rho_{AY}$, and the density of the Na-β”-alumina is $\rho_{\beta {}^{\prime \prime }Y}$. The initial mass of the sample is

(A10) $$m_{o}=\rho_{AY}V_{o}=\rho_{AY}Al_{o}$$

After time t, the thickness $x{}^{\prime }(t)$ of the initial sample on both surfaces converts to Na-β”-alumina + YSZ of thickness x(t). Thus, the sample thickness at time t becomes

(A11) $$l(t)=l_{o}+2x(t)-2x{}^{\prime }(t)$$

and the volume at time t is

(A12) $$V(t)=Al(t)=A\left(l_{o}+2x(t)-2x{}^{\prime }(t)\right)$$

The corresponding mass at time t is

(A13) $$m(t)=A\left\{\rho_{AY}\left(l_{o}-2x{}^{\prime }(t)\right)+\rho_{\beta {}^{\prime \prime }Y}2x(t)\right\}=\rho_{AY}Al_{o}+2A\left(x(t)\rho_{\beta {}^{\prime \prime }Y}-x{}^{\prime }(t)\rho_{AY}\right)$$

or

(A14) $$m(t)=m_{o}+\Delta m(t)$$

where

(A15) $$\Delta m(t)=2A\left(x(t)\rho_{\beta {}^{\prime \prime }Y}-x{}^{\prime }(t)\rho_{AY}\right)$$

is the increase in sample weight after time t. Note that

(A16) $$\frac{x{}^{\prime }(t)}{x(t)}=\frac{l_{o}}{l(\infty )}=\frac{m_{o}\rho_{\beta {}^{\prime \prime }Y}}{m(\infty )\rho_{AY}}$$

where the final thickness after full conversion is l(∞), and the final mass is m(∞).

Thus,

(A17) $$\Delta m(t)=2Ax(t)\rho_{\beta {}^{\prime \prime }Y}\left(1-\frac{m_{o}}{m(\infty )}\right)=2Ax(t)\rho_{\beta {}^{\prime \prime }Y}\left(1-\frac{\rho_{AY}l_{o}}{\rho_{\beta {}^{\prime \prime }Y}l(\infty )}\right)$$

The $\rho_{AY}$, $l_{o}$, and $m_{o}$ are measured on the initial sample. The $\rho_{\beta {}^{\prime \prime }Y}$ is measured on a fully converted sample. Thus, both l(∞) and m(∞) can be estimated. The preceding implies that it is not necessary to carry out full conversion to achieve the final weight, m(∞), and the final thickness, l(∞), in order to use the kinetic equation given in (A1). In the present work, however, the sample was heat-treated for a long enough time to achieve the final weight and thickness. Thus, Equation (A17) is used in the analysis.

From Equation (A17), the conversion thickness in terms of weight gain is given by

(A18) $$x(t)=\frac{\Delta m(t)}{2A\rho_{\beta {}^{\prime \prime }Y}\left(1-\frac{m_{o}}{m(\infty )}\right)}=\frac{\Delta m(t)}{2A\rho_{\beta {}^{\prime \prime }Y}\left(1-\frac{\rho_{AY}l_{o}}{\rho_{\beta {}^{\prime \prime }Y}l(\infty )}\right)}$$

Equations (A17) and (A18) show that the conversion thickness x(t) at time t can be given in terms of change in weight, Δm(t), and the densities of the initial sample, ($\rho_{AY}$), and after conversion to Na-β”-alumina, ($\rho_{\beta {}^{\prime \prime }Y}$), the sample cross sectional area, A; the initial and final sample thicknesses, $l_{o}$ and l(∞), respectively; and the initial and final masses, $m_{o}$ and m(∞), respectively. Therefore, Equation (A1) can be written as

(A19) $$\frac{{\left(\Delta m(t)\right)}^{2}}{4D_{eff}{\left(A\rho_{\beta {}^{\prime \prime }Y}\left(1-\frac{m_{o}}{m(\infty )}\right)\right)}^{2}}+\frac{\Delta m(t)}{2k_{eff}\left(A\rho_{\beta {}^{\prime \prime }Y}\left(1-\frac{m_{o}}{m(\infty )}\right)\right)}=t$$

Thus, by measuring Δm(t) as a function of time t and fitting experimental data to Equation (A19), it should be possible to obtain both kinetic parameters, $D_{eff}$ and $k_{eff}$. Alternatively, Equation (A19) may be linearized as follows

(A20) $$\Delta m(t)=4A^{2}{\left(\rho_{\beta {}^{\prime \prime }Y}-\rho_{AY}\lambda \right)}^{2}D_{eff}\frac{t}{\Delta m(t)}-2A(\rho_{\beta {}^{\prime \prime }Y}-\rho_{AY}\lambda )\frac{D_{eff}}{k_{eff}}$$

A plot of Δm(t) versus $\frac{t}{\Delta m(t)}$ is thus expected to be linear. Then, from the slope and the intercept, it should be possible to obtain both $D_{eff}$ and $k_{eff}$.
